# Alterations of Striato-Thalamic Metabolism in Normal Aging Human Brain—An MR Metabolic Imaging Study

**DOI:** 10.3390/metabo11060371

**Published:** 2021-06-09

**Authors:** Mareike Ahlswede, Patrick Nösel, Andrew A. Maudsley, Sulaiman Sheriff, Nima Mahmoudi, Paul Bronzlik, Heinrich Lanfermann, Xiao-Qi Ding

**Affiliations:** 1 Institute of Diagnostic and Interventional Neuroradiology, Hannover Medical School, Carl-Neuberg-Str. 1, 30625 Hannover, Germany; Ahlswede.Mareike@mh-hannover.de (M.A.); Noesel.Patrick@mh-hannover.de (P.N.); Mahmoudi.Nima@mh-hannover.de (N.M.); Bronzlik.Paul@mh-hannover.de (P.B.); Lanfermann.Heinrich@mh-hannover.de (H.L.); 2 Department of Radiology, University of Miami School of Medicine, Miami, FL 33136, USA; AMaudsley@med.miami.edu (A.A.M.); SSheriff@med.miami.edu (S.S.)

**Keywords:** whole brain ^1^H-MR spectroscopic imaging, aging human brain, metabolic alteration, basal ganglia, thalamus

## Abstract

Aging effects on striato-thalamic metabolism in healthy human brains were studied in vivo using short-TE whole brain ^1^H-MR spectroscopic imaging (wbMRSI) on eighty healthy subjects aged evenly between 20 to 70 years at 3T. Relative concentrations of N-acetyl-aspartate (NAA), choline, total creatine (tCr), myo-inositol (mI), glutamate, and glutamine in bilateral caudate nucleus, putamen, pallidum, and thalamus were determined using signal normalization relative to brain tissue water. Linear regression analysis was used to analyze the age-dependence of the metabolite concentrations. The metabolite concentrations revealed spatial inhomogeneity across brain regions and metabolites. With age, NAA decreased significantly in bilateral caudate nucleus and putamen, left pallidum, and left thalamus, tCr decreased in left putamen and bilateral pallidum, mI increased in bilateral caudate nucleus and right thalamus, and spectral linewidth increased in left putamen and right thalamus. In conclusion, normal aging of striato-thalamic metabolism in healthy human is associated with regional specific decreases of NAA and tCr and increases of mI, which may reflect the individual role of each brain structure within brain functionality.

## 1. Introduction

Normal aging of the human brain is characterized by decreased cognitive and motor performance, which directly impact the quality of life and increase vulnerability to neurodegenerative diseases [1,2]. Correspondingly, in a society with an increasing life expectancy, research on physiological aging in healthy human brain becomes more important and to investigate procedures for healthy aging and early identification of neurodegenerative changes. Estimation of age-related changes of brain metabolite concentrations, detected using ^1^H magnetic resonance spectroscopy (^1^H-MRS), could provide information about age-related altered brain metabolism, because the ^1^H-MRS-accessible chemicals of N-acetyl-aspartate (NAA), choline (Cho), total creatine (tCr), myo-inositol (mI), glutamine (Gln), and glutamate (Glu) are brain metabolites related to neurometabolic activity and integrity. The largest signal in the normal adult brain spectrum is NAA, which is considered to be a marker for the assessment of brain neuronal integrity, although its exact functions remain under investigation [3]. The Cho signal includes contributions from several choline-containing compounds that are involved in membrane synthesis and degradation and is therefore considered to be a marker for altered membrane turnover. tCr is considered as a marker for energy metabolism since it contains both creatine and phosphocreatine that are involved in energy metabolism. mI is involved with osmoregulation and is a marker of the glial cell population and reflects gliosis. Glu is the dominant excitatory neurotransmitter, and Gln is involved in recycle of Glu between neurons and astrocytes.

**Figure 1:**
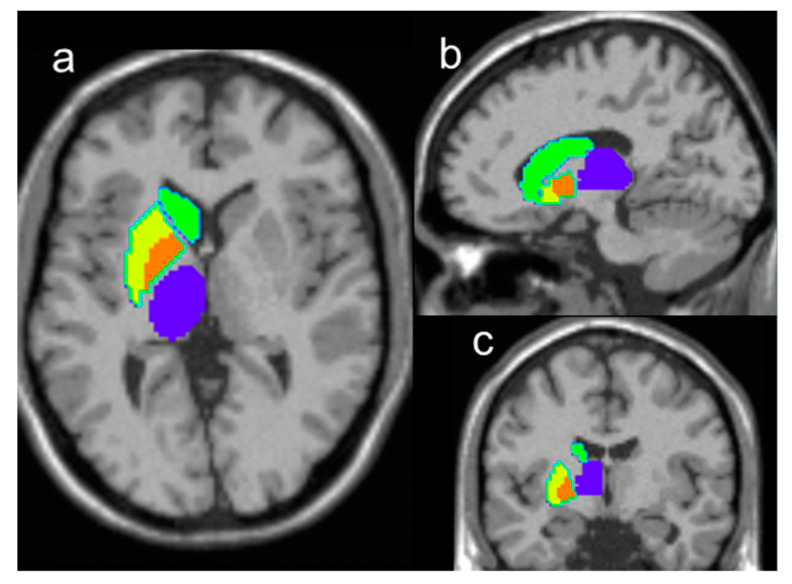
Illustration of the AAL-atlas defined regions used for the analysis, including right caudate nucleus (green filled areas), putamen (yellow filled areas), pallidum (orange filled areas), and thalamus (blue filled areas), overlaid on T1 weighted images in traverse section (**a**), sagittal section (**b**), and coronal section (**c**), and corresponding regions in the left hemisphere (without colored marker).

To date, numerous ^1^H-MRS studies on aging brains were reported; however, due to limited spatial coverage of the standard data acquisition techniques, most of these studies were carried out on only a few small brain regions, selected prior to the MR examinations (Haga et al., 2009). By using a whole brain ^1^H-MR spectroscopic imaging (wbMRSI) acquisition technique, better spatial sampling can be achieved within an acceptable scan time and with sufficient spatial resolution for human studies [4,5]. It was demonstrated that the wbMRSI technique could be used to measure metabolite contents over multiple large brain regions, such as brain lobes [6,7], or in multiple specific brain regions [8,9] simultaneously. In combination with advanced analysis methods, it is now possible to measure metabolite contents from specific brain structures, segmented according to a brain atlas, as well as measure metabolites that can otherwise be difficult to differentiate, such as separate measurement of Glu and Gln [10]. In this study, these methods were used to measure the relative concentrations of brain metabolites in bilateral caudate nucleus, putamen, pallidum, and thalamus of striato-thalamic regions (Figure 1) of healthy subjects, whose lobar metabolic changes with age were reported previously [7], for the purpose of investigating normal aging associated alterations of the striato-thalamic metabolism. This study demonstrates the advantages provided by the wbMRSI technique, i.e., the freedom in analyzing metabolic changes in multiple associated brain regions chosen according to a specific purpose. These brain structures were selected because of their essential role in brain functions and their relevance for research on neurodegenerative diseases.

## 2. Results

**Figure 2:**
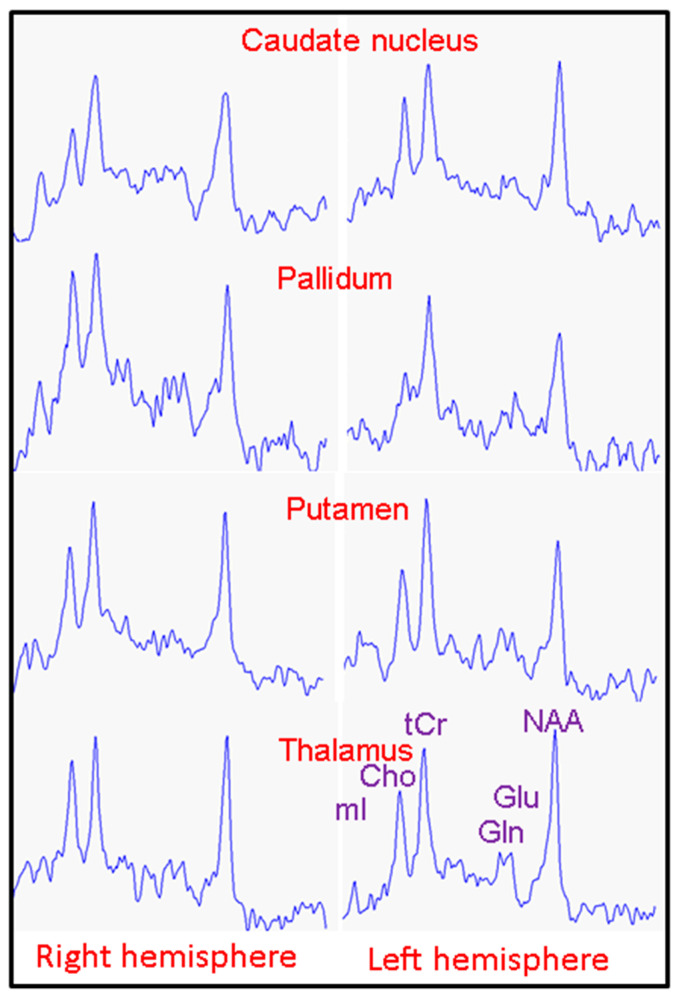
Example MR spectra obtained following averaging of multiple wbMRSI voxels over right and left caudate nucleus, putamen, pallidum, and thalamus from a 31-year-old male subject, from which the concentrations of the metabolites NAA, Cho, tCr, Glu, Gln, and mI of the brain structures were determined in spectral analysis.

**Figure 3:**
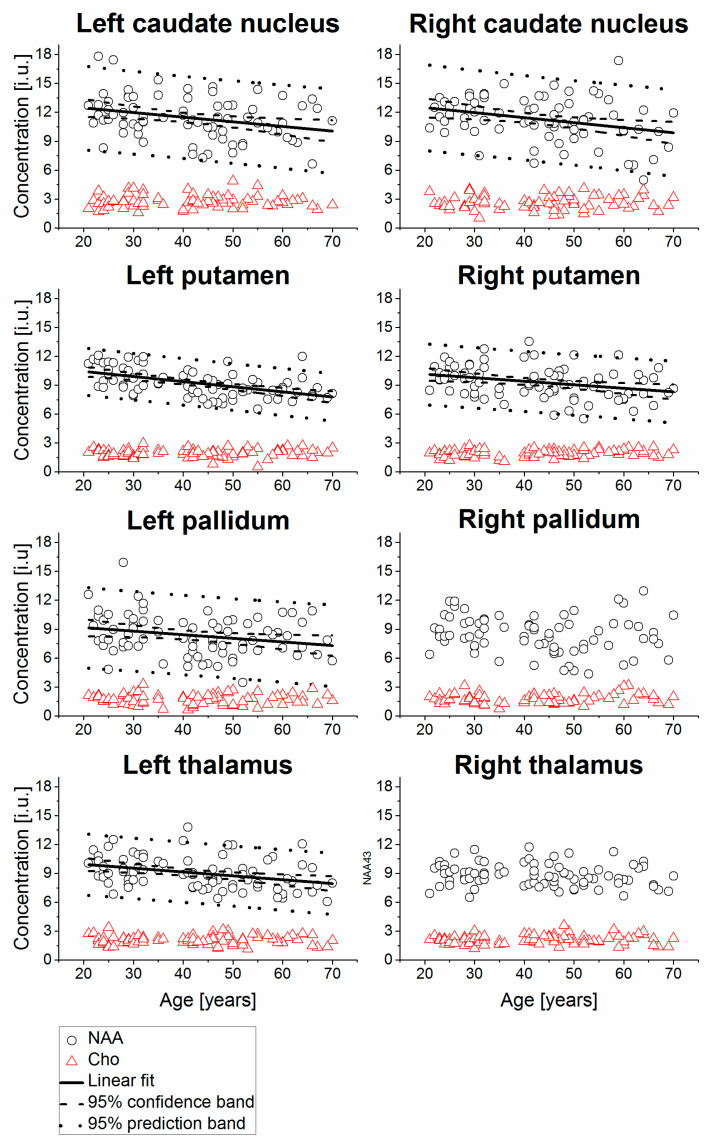
Relative concentrations of NAA and Cho from bilateral caudate nucleus, putamen, pallidum, and thalamus shown as a function of age, including linear fits with 95% prediction interval and 95% confidence interval for statistically significant cases (*p* < 0.05). The values are presented in institutional units (i.u.) normalized using the signal from tissue water.

**Figure 4:**
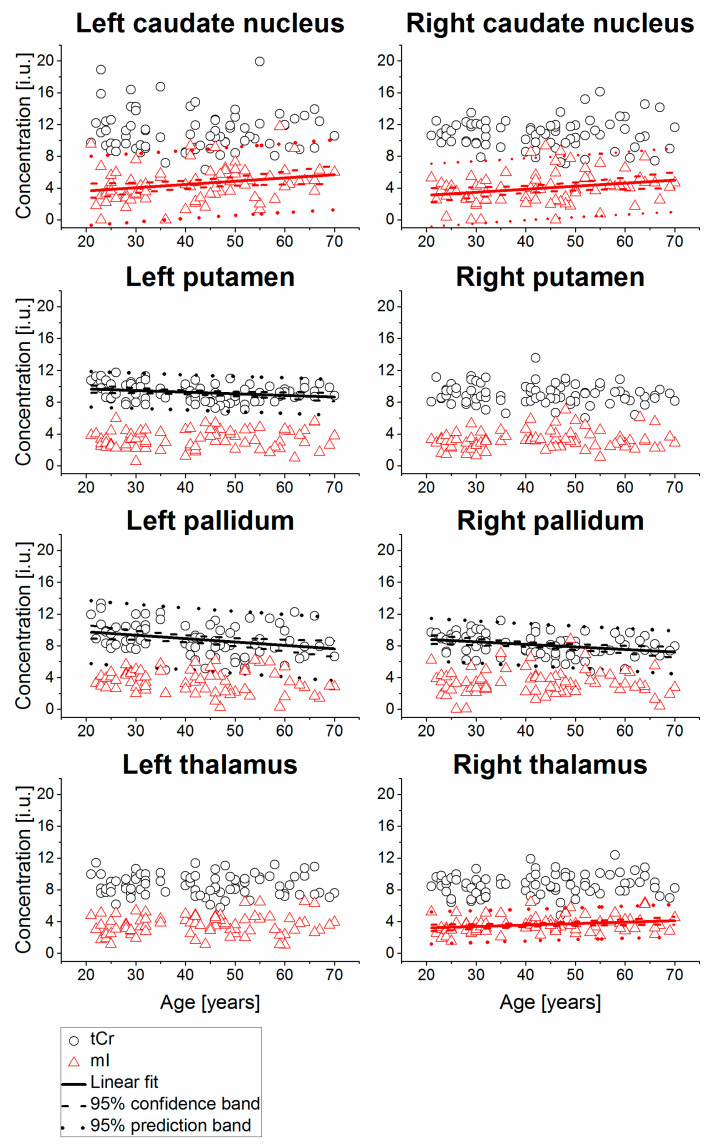
Relative concentrations of tCr and mI from bilateral caudate nucleus, putamen, pallidum, and thalamus, shown as a function of age, including linear fits with 95% prediction interval and 95% confidence interval for statistically significant cases (*p* < 0.05). The values are presented in institutional unit (i.u.) normalized using the signal from tissue water.

**Figure 5:**
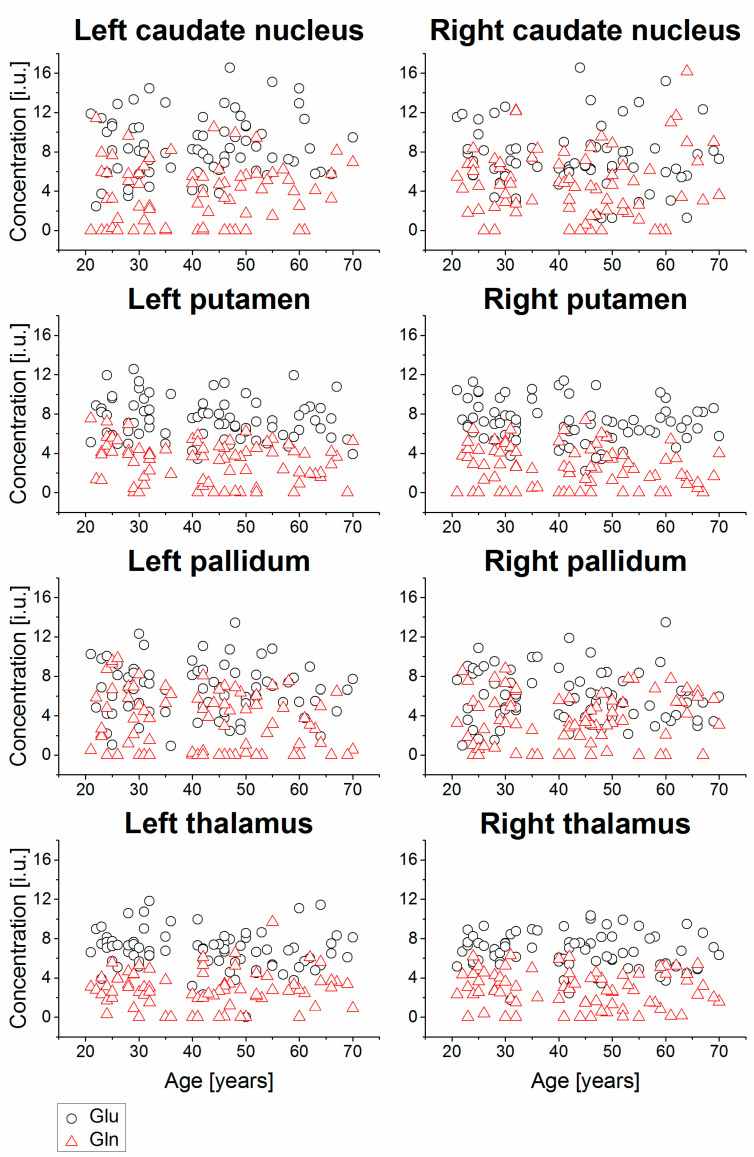
Relative concentrations of Glu and Gln from bilateral caudate nucleus, putamen, pallidum, and thalamus of all subjects, shown as a function of age. The values are presented in institutional unit (i.u.) normalized using the signal from tissue water.

**Figure 6:**
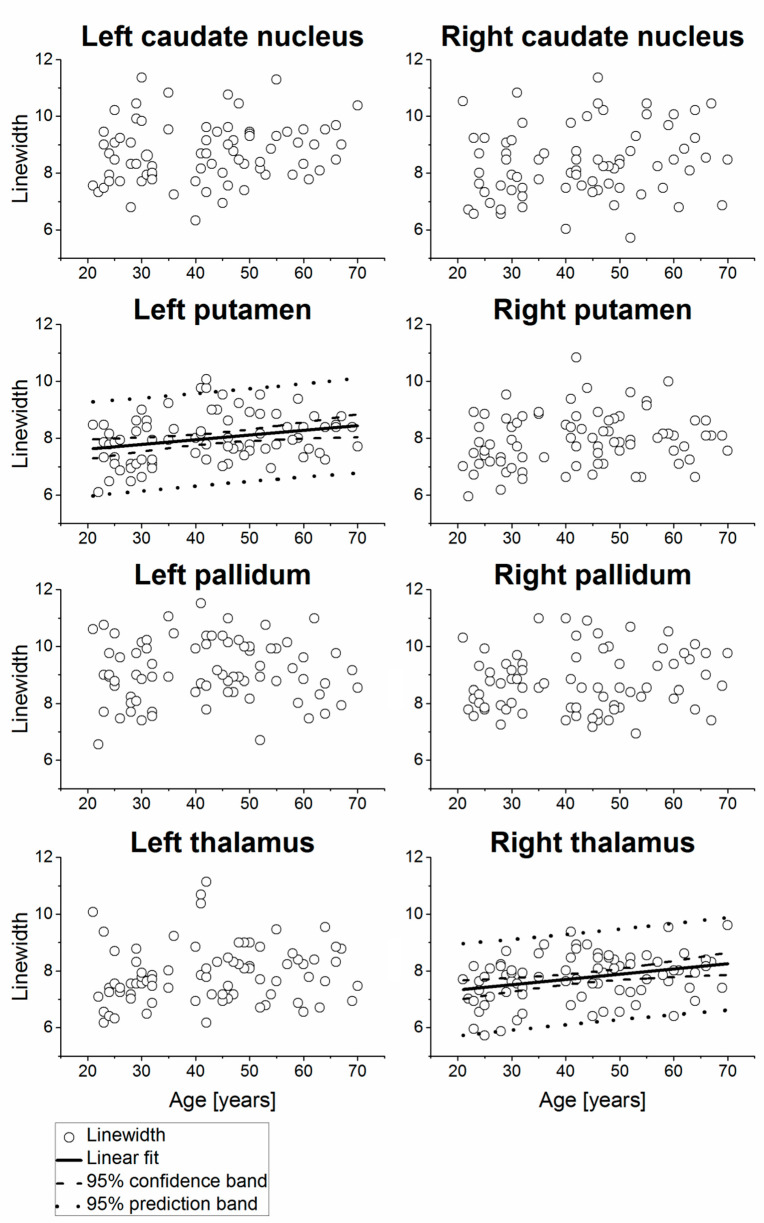
Spectral linewidths of the subjects measured from bilateral caudate nucleus, putamen, pallidum, and thalamus of the subjects, shown as a function of age, including linear fits with 95% prediction interval and 95% confidence interval for statistically significant cases (*p* < 0.05). The values are presented in Hz.

Example MR spectra obtained following averaging of multiple wbMRSI voxels over right and left caudate nucleus, putamen, pallidum, and thalamus are shown in Figure 2, which were obtained from a 31-year-old male subject. In Figure 3, Figure 4, Figure 5 and Figure 6 are shown the values of the metabolite concentrations and the spectral linewidths from bilateral caudate nucleus, putamen, pallidum, and thalamus, respectively, according to age and including linear fits with 95% prediction interval and 95% confidence interval for statistically significant cases (*p* < 0.05). The mean values of regional metabolite concentrations and the spectral linewidth in each of the eight brain areas as well as the results of the linear regression analysis are given in Table 1. The results indicate that the mean metabolite concentrations of NAA, Cho, tCr, Glu, Gln, and mI varied among caudate nucleus, putamen, pallidum, and thalamus in both hemispheres, while hemispheric differences between regions were small. Significant (*p* < 0.05) decreases with age were found for (NAA) in six of eight regions, i.e., in bilateral caudate nucleus and putamen and in left pallidum and thalamus, with the coefficient R varying from −0.52 to −0.24 and a decreasing rate from −3.7% to −5.3% per decade, and for (tCr) in three of eight regions (left putamen and bilateral pallidum, with R varying from −0.33 to −0.25 and a decreasing rate from −2.0% to −4.3% per decade). Significant increases with age were found for (mI) in three of eight regions (bilateral caudate nucleus and right thalamus, R = 0.26, 0.25, and 0.25, respectively) with rates varying from 1.8% to 4.1% per decade as well as for spectral linewidth in left putamen and right thalamus (R = 0.21 and 0.37) with rates of 1.7% and 1.8% per decade, respectively.

## 3. Discussion

The putamen forms, with the globus pallidus, the lentiform nucleus, and with the caudate nucleus, the striatum. Altogether, these comprise the basal ganglia. Together with the thalamus, these brain structures play essential roles in brain function, although the primary functions of each brain structure are not yet fully clarified. It is known that the striatum is the major input source of the basal ganglia, which receives inputs from the cerebral cortex, and the efferent projections processed in the striatum travel to the hippocampus and the globus pallidus and return to the cerebral cortex via the thalamus. Dysfunctions in these brain structures found in various motor and cognitive dysfunctions associated with neurodegenerative or mental disorders [11,12,13]. Therefore, knowledge about the metabolic changes with age in these brain structures may help to understand their functionality. However, to date, metabolite concentrations in the individual structures of the human basal ganglia are rarely reported.

Numerous brain MRS studies of aging in human were published, with most reporting measurements of NAA, Cho, tCr, and a few including Glx (mixture of Glu and Gln) and mI, while the measurements were mainly done by considering basal ganglia as one brain region [14,15]. The main reason for the shortcoming could be that these studies used the vendor provided conventional MRS techniques that are restricted in the number of regions that can be sampled and the shapes of those regions. These weaknesses limit the placement of ROIs in individual structures of basal ganglia and are not well suited for discrimination of Glu and Gln.

In this study, based on the data acquired with wbMRSI, brain metabolite concentrations were determined in bilateral caudate nucleus, putamen, pallidum, and thalamus of the striato-thalamic regions that were defined according to a brain atlas. The choice to separately determine Glu and Gln was based on several reasons. Even though it was shown that spectral analysis using Glx values results in lower CRLB values than individual fitting of Glu and Gln [10], as this study may serve as reference data for patient studies, and the basal ganglia region is sensitive to cognitive changes occurring with age and neurodegeneration, the separate detection of Glu and Gln may provide additional information, especially because the ratio of Glu/Gln is known to vary between brain areas [10]. Up to date, comparable studies determining Glu and Gln separately are rarely reported. To estimate our data quality for separating Glu and Gln, we calculated the correlations of measured Glu and Gln concentrations at each brain region. As shown in the method section about quality assessment of the spectral data, the correlation coefficient R showed a value mostly around −0.5, except in right pallidum (R = −0.7). Considering that, if Glu and Gln are impossible to separate by spectral fitting, the R would be close to −1, our results with a value about −0.5 indicated the possibility of separating Glu and Gln in these brain structures, while the potential inter-dependence of the fitting of Glu and Gln is a source of error. Therefore, the obtained Glu and Gln values should be interpreted with caution, especially those measured in right pallidum. Further studies to verify the results are needed.

While averaging of individual spectra over anatomic regions can improve detection of resonances from mI, Glu, and Gln, the ROIs selected for this study were relatively small. The adapted selection criteria for CRLB values of mI, Glu, and Gln ((mean absolute CRLB) ± 2 times standard deviations) suggested by Kreis were applied. This choice was made because simply thresholding the final CRLB values may bias data selection, and small metabolite levels may not be detected [16].

As shown in Table 1, the derived mean (NAA), (Cho), (tCr), (Glu), (Gln), and (mI) were mostly comparable to those reported previously for human brain by using conventional MRS techniques [10,11]. To our knowledge, this study is the first to simultaneously determine individual concentrations of NAA, Cho, tCr, Glu, Gln, and mI in four brain regions, namely caudate nucleus, putamen, pallidum, and thalamus of human brain, and demonstrates the advantage of using the advanced metabolic imaging method. As shown in Table 1 and Figure 2, Figure 3 and Figure 4, the results indicate spatial inhomogeneity across these brain regions, which differ by metabolite. For example, the mean (NAA) was highest in bilateral caudate nucleus (11.3–11.4 i.u.), then in putamen (9.2–9.3 i.u.), thalamus (8.8–9.0 i.u.), and lowest in pallidum (8.3–8.4 i.u.). The mean (Cho) revealed a similar variation but slightly different in the regional order, with highest values also in bilateral caudate nucleus (2.6–2.8 i.u.), then in thalamus (~2.1 i.u.), putamen (~1.95 i.u.), and lowest in pallidum (1.7–1.8 i.u.). The mean (Glu), (Gln), and (mI) also showed some regional differences, but not in such a clearly ordered regional arrangement, except that all the means were the highest in bilateral caudate nucleus. The small hemispheric differences between regions in some of the metabolite findings may be attributed to the difference in the number of voxels or to the handedness. Further study on subjects also with balanced handedness distribution is needed to verify it.

A further finding of this study was that striato-thalamic metabolites changed with age. Age-related cerebral metabolite changes in healthy subjects were previously reported [14,15], though few of these studies reported on metabolite changes in basal ganglia, lentiform nucleus, or thalamus. Our findings of the decreased (NAA) with age in bilateral caudate nucleus and putamen and in left pallidum and thalamus may be interpreted as age-related reduced neuronal density or neuronal function in these brain areas [17,18]. The decreased (tCr) in bilateral pallidum and left putamen suggest altered energy metabolism, the unchanged (Cho) in all measured structures as constant cell membrane turnover in striato-thalamic regions [19,20]. These findings are consistent with those reported in previous studies on age-related changes in basal ganglia, e.g., a decrease of (NAA) in basal ganglia was found by numerous previously reported studies [21,22,23,24], a decrease of (tCr) was reported by Charles et al. [22] and Zahr et al. [24], and unchanged (Cho) with age was reported by Harada et al. [21]. Up to now, age-related changes of metabolites Glu, Gln, and mI determined in basal ganglia or thalamus were hardly reported. We also found that (mI) increased with age in bilateral caudate nucleus and in right thalamus, while an increased (mI) was reported in cortical gray matter [25], thalamus (higher level in in the middle than the younger group) [26], and cerebral white matter [23,27], which was considered an indication for gliosis. However, there were findings that showed discrepancies with our results. While decreased (NAA) with age was frequently reported by previous studies, the results concerning (Cho) and (tCr) were less consistent, as were rarely reported age-related changes of Glu, Gln, and mI in strato-thalamic regions. For example, in the lentiform nucleus, Harada et al. found no age-related changes of tCr [21], and Charles et al. reported decreased (Cho) with age [22], while Gruber et al. reported no age-related changes of NAA, Cho, and tCr in thalamus [23], whereas Zahr et al. found decreased striatal (Glu) with age [24], which are all different from our corresponding findings. These differences could be caused by multiple factors, such as the composition of subjects included in the studies, e.g., sample size, age distribution, health status, and MRS techniques used, including that most of the previously reported studies targeted brain areas quite differently from the method used in this study.

In agreement with previous reports, this study observed increased spectral linewidth with age, which was significant in the left putamen and the right thalamus (Figure 5). This observation can be interpreted as a regionally dependent increase in brain iron concentrations and corresponding shorter metabolite transverse relaxation times in older subjects [28,29,30]. Eylers et al. found decreased T2’, i.e., the transverse relaxation time that is related to local magnetic field inhomogeneity, and decreased NAA concentration and tCr concentration with age in putamen and pallidum, where T2’ was considered a marker for iron deposition, and the findings were interpreted as a combined effect of increased iron deposition, decreased neuronal activity, or function and decreased energy metabolism [8]. As reviewed by Ashraf et al., iron concentration was shown to increase with age, with highest values detected in the basal ganglia. Due to the association with oxidative cell damage and a decrease of cognitive functions, iron deposition is considered as a marker for cognitive decline in normal aging [31]. Additionally, abnormal increased iron concentration is also a hallmark of neurodegenerative diseases such as Parkinson’s disease or Alzheimer’s disease [29]. Therefore, the combination of alterations in metabolite concentrations and iron deposition in basal ganglia may explain why this region is especially susceptible to effects of aging and neurodegeneration. An increase of spectral linewidth might also result in an increased overlap between neighboring resonances, thereby affecting the accuracy of the metabolite concentration estimates. However, since the linewidths were all within a relatively narrow range (≤9.13 ± 1.22 Hz), and the observed changes with age were within 1.8%, it is unlikely this had significant impact on the estimation of these metabolite concentrations.

It is interesting to note that the measurements of individual brain structures allowed us to estimate spatial distribution of the brain metabolites as well as their age-dependent changes. As seen in Table 1, spatial inhomogeneity within striato-thalamic regions was found not only for measured regional metabolite concentrations but also for their changes with age. These spatial metabolic differences may reflect the individual roles of caudate nucleus, putamen, pallidum, and thalamus for brain functionality. Our data may contribute to understanding the basic functions of the brain structures as well as provide reference data for detection of striato-thalamic metabolic changes under different pathological conditions in patients.

Limitations of this study include that the signal normalization did not account for possible differences in water or metabolite relaxation rates between subjects or with age. Possible effect of handedness was not considered because of limited sample size (*n* = 7 for left handedness), and subjects younger than 20 and older than 70 were not included due to difficulties in subject recruitment. Moreover, metabolite concentrations were not reported in absolute concentrations due to a missing external reference and results in increased variability of metabolite ratios across subjects [9]. This reference may be separately acquired but often prolongs the acquisition time being unavailable in clinical measurements. A related limitation is that the signal normalization did not account for possible differences in brain tissue water or metabolite relaxation rates between subjects or with age. Additionally, the rather small ROIs may have impacted the determination of metabolites with small signals such as Glu or Gln. A study with a larger sample is needed to verify the results.

## 4. Materials and Methods

### 4.1. Subjects

The study was approved by the local Institution Review Board and conducted according to the principles expressed in the Declaration of Helsinki. Written consent was obtained from all subjects before the examinations. All subjects were registered in an existing database for brain studies at our institute. The participants were recruited from the local population and interviewed to exclude any history of brain disease or injury or cognitive or mental impairments. Each subject underwent two screening tests (DemTect and BDI II) to exclude cognitive or psychiatric impairments [32,33]. Subjects with abnormal results of screening tests, incomplete MR examinations, and excess body weight (body mass index ≥ 30) or brain morphological alterations were excluded. Eighty of 81 healthy subjects, whose data were acquired between 2012 and 2015 and included in a previous study to determine brain lobar metabolite concentrations in aging humans [7], were included. One subject (male, 69 years old) was excluded due to an abnormal DemTect score found later. The age of the subjects was distributed evenly between 20 to 70 years (46 females and 34 males, mean age 43 ± 14 years, *n* > 12 subjects with at least 5 males and 5 females per decade).

### 4.2. MR Examination and Data Processing

All subjects took part in an MR examination at 3T (Verio, Siemens, Erlangen, Germany) with a twelve-channel phased-array receive-only head coil. The MRI scan protocol included a T1-weighted 3D MPRAGE (magnetization prepared rapid gradient echo) acquisition at 1 mm isotropic resolution and a volumetric spin-echo planar spectroscopic imaging (EPSI) sequence (TR/TE = 1550/17.6 ms, 50 × 50 voxels in-plane and 18 slices, over a field-of-view of 280 × 280 × 180 mm^3^) with parallel imaging acquisition and GRAPPA reconstruction for wbMRSI. The acquisition included a second dataset obtained without water suppression that was used for several processing functions and normalization of the metabolite concentrations as described previously [34].

Metabolite image reconstruction was carried out for data acquired with the EPSI sequence using the MIDAS (metabolic imaging and data analysis system) software package [35,36]. The data processing included correction of B0 shifts, creation of white-matter, gray-matter, and cerebrospinal fluid (CSF) segmentation maps using SPM software (https://www.fil.ion.ucl.ac.uk/spm/software/spm12, accessed on 6 June 2021), and affine registration between the T1-weighted MRI and whole-brain MRSI. The metabolite maps were interpolated to 64 × 64 × 32 points with an interpolated voxel volume of 0.107 cm^3^. Following spatial smoothing, the effective voxel volume was 1.55 cm^3^. For spectral analysis, the approach introduced by Goryawala et al. was used [10], in which the spectra were averaged (integrated spectra) by summing voxels within each selected region of interest (ROI), i.e., in caudate nucleus, putamen, pallidum, and thalamus in each hemisphere, respectively, to obtain spectra with a high signal to noise ratio (SNR). All striato-thalamic regions were defined in subject space using inverse spatial transformation of a spatial reference brain defined in MNI space [37], which was associated with a modified version of the automated anatomical labeling (AAL) atlas [10,38]. Partial volume contributions associated with summing voxels within each brain area for spectral averaging was reduced by thresholding the volume contribution of the selected ROI at a level of 50%. Prior to averaging, spectral analysis of individual voxels was carried out that included correcting the spectra for frequency and phase variation and determination of the linewidths for the metabolite resonances [39]. Voxels showing a spectrum with a linewidth greater than 10 Hz or a CSF fraction volume (FVCSF) greater than 20% were then excluded from further analysis. Following inverse spatial transformation of the atlas into subject space, the integrated spectra of each brain area were created. The mean numbers of voxels averaged were 13 and 14 voxels for left and right caudate nucleus, 33 and 37 voxels for left and right putamen, 8 and 6 voxels for left and right pallidum, and 36 voxels for the thalamus. All integrated spectra underwent spectral analysis by using functionality provided in the MIDAS package to estimate relative concentrations of the metabolites, which were determined as a ratio to the corresponding signal from the water MRSI corrected for the assumed water content of 79% [36], denoted as (NAA), (Cho), (tCr), (Glu), (Gln), and (mI) and presented in institutional unit (i.u.). The i.u. was used because the measurements of exact metabolite concentrations required additional calibration measurements that are impractical for in vivo studies. The corresponding spectral linewidth were also derived.

### 4.3. Quality Assessment of the Spectral Data

The results of the spectral analysis were controlled by the following quality criteria: those were excluded from further analysis if spectral fitting showed an uncertainty as measured by relative Cramér–Rao lower bounds (CRLBs) exceeding either 20% (for NAA, Cho, tCr) or the range of (mean CRLB) ± 2 times standard deviations (SD) to avoid the bias of removing smaller concentration values (for Glu, Gln, mI) [16,40]. The mean absolute CRLBs of mI, Gln, and Glu together with their SDs are also shown (Table 2) to provide an overview about the data quality concerning smaller concentration values. For a quality estimation of separating Glu and Gln, the correlation of derived Glu and Gln concentrations of each brain structure was calculated with Pearson’s correlation test (Table 3).

### 4.4. Statistical Analysis

The normal distribution of the data was proven with Shapiro–Wilk test and quantile-quantile plots. In addition, one or two metabolite values of each brain structure might be removed as outliers estimated by use of explorative analysis. A two-sided *t*-test was used to test the hypothesis that the mean metabolite concentrations of the metabolites for males and females in each of the eight brain regions were equal, which revealed only very few data showing a gender difference (*p* < 0.05 for Glu and mI in left putamen, mI in left pallidum; altogether ≤6% datasets). Therefore, to improve the power of the statistical analysis, the data of males and females were all combined and used in the following analysis. One-way analysis of variance (ANOVA) for trend analysis was used to estimate the relationship between metabolite concentrations and the age. Where a significant linear correlation was found, linear regression analysis was then used to estimate the age dependencies of the regional metabolite concentrations and the spectral linewidth derived from each brain area. A repeated measure of ANOVA with a Greenhouse–Geisser correction determined that mean metabolite values showed a statistically significant difference between 8 brain structures, and the post hoc pairwise tests revealed that the means of main metabolites NAA, Cho, and tCr were mostly significantly different between the brain structures. Therefore, when testing for individual age-related metabolic changes in each of the selected brain structures, the data derived from bilateral caudate nucleus, putamen, pallidum, and thalamus were analyzed independently from each other with a significance level of 0.05. Statistical analyses were performed with SPSS version 24 (SPSS IBM, New York, NY, USA), and the graphic illustrations were done with the software OriginPro (OriginLab, Northampton, MA, USA).

## 5. Conclusions

Human brain metabolite concentrations and their age-dependences revealed spatial inhomogeneity among caudate nucleus, putamen, pallidum, and thalamus; the normal aging of striato-thalamic metabolism in healthy human is associated with regional specific decreases of NAA and tCr concentrations and increases of mI concertation. This study demonstrates advantages of the wbMRSI method for in vivo metabolic imaging studies of the human brain.

## Figures and Tables

**Table 1:** The mean values and the age associations of the regional metabolite concentrations and the spectral linewidths estimated with linear regression analysis.

Region	NAA									tCr							
	N ^a^	Mean ^b^	SD ^b^	R	*p*	Intercept ^b^	Slope ^b^		Variations ^c^	N ^a^	Mean ^b^	SD ^b^	R	*p*	Intercept ^b^	Slope ^b^	Variations ^c^
**Right caudate nucleus**	**74**	**11.28**	**2.28**	**−0.31**	**0.0072**	13.53 ± 0.85	−0.0523 ± 0.0189		−5.2	74	10.82	1.83	0.02	0.8830			
**Left caudate nucleus**	**76**	**11.40**	**2.21**	**−0.29**	**0.0103**	13.41 ± 0.80	−0.0482 ± 0.0183		−4.8	76	11.13	2.55	−0.02	0.8378			
**Right putamen**	**80**	**9.30**	**1.63**	**−0.31**	**0.0050**	10.87 ± 0.57	−0.0367 ± 0.0127		−3.7	80	9.01	1.25	−0.12	0.2910			
**Left putamen**	**80**	**9.22**	**1.40**	**−0.52**	**0.0000**	11.48 ± 0.44	−0.0531 ± 0.0098		−5.3	**80**	**9.19**	**1.13**	**−0.25**	**0.0254**	10.07 ± 0.40	−0.0205 ± 0.0090	−2.0
**Right pallidum**	75	8.41	1.91	−0.16	0.1819					**75**	**8.09**	**1.38**	**−0.33**	**0.0044**	9.45 ± 0.49	−0.0322 ± 0.0110	−3.2
**Left pallidum**	**77**	**8.33**	**2.11**	**−0.24**	**0.0330**	9.91 ± 0.76	−0.0374 ± 0.0172		−3.7	**77**	**8.80**	**2.02**	**−0.29**	**0.0102**	10.61 ± 0.72	−0.0428 ± 0.0162	−4.3
**Right thalamus**	80	8.82	1.17	−0.16	0.1534					80	8.63	1.33	0.11	0.3333			
**Left thalamus**	**80**	**9.02**	**1.64**	**−0.34**	**0.0022**	10.73 ± 0.57	−0.0400 ± 0.0127		−4.0	80	8.63	1.30	−0.04	0.7362			
**Region**	**Cho**							**Glu**					**Gln**				
	**N ^a^**		**Mean ^b^**		**SD ^b^**	**R**	***p***	**N ^a^**	**Mean ^b^**	**SD ^b^**	**R**	***p***	**N ^a^**	**Mean ^b^**	**SD ^b^**	**R**	***p***
**Right caudate nucleus**	74		2.61		0.70	0.07	0.5784	72	7.20	3.16	−0.07	0.5483	72	4.72	3.46	0.06	0.6276
**Left caudate nucleus**	75		2.77		0.66	0.00	0.9856	70	8.59	3.09	0.10	0.4282	73	3.69	3.12	0.08	0.5064
**Right putamen**	79		1.95		0.38	0.15	0.1905	75	7.15	2.07	−0.20	0.0915	78	2.53	2.05	−0.18	0.1113
**Left putamen**	80		1.93		0.41	0.02	0.8555	76	7.53	2.05	−0.19	0.1076	77	3.08	2.07	−0.19	0.0901
**Right pallidum**	75		1.76		0.50	0.13	0.2689	74	5.90	2.63	−0.11	0.3585	71	3.64	2.59	0.08	0.5121
**Left pallidum**	77		1.71		0.53	0.00	0.9845	73	6.67	2.71	−0.07	0.5825	76	3.46	3.00	−0.21	0.0740
**Right thalamus**	80		2.11		0.45	0.06	0.6059	75	6.68	1.80	−0.08	0.4979	76	2.72	1.83	−0.11	0.3600
**Left thalamus**	80		2.09		0.46	−0.02	0.8782	78	6.81	2.01	−0.16	0.1613	76	2.79	1.89	0.04	0.7213
**Region**	**mI**									**Linewidth (Hz)**							
	**N ^a^**	**Mean ^b^**	**SD ^b^**	**R**	***p***	**Intercept ^b^**	**Slope ^b^**		**Variations ^c^**	**N ^a^**	**Mean**	**SD**	**R**	***p***	**Intercept ^b^**	**Slope ^b^**	**Variations ^c^**
**Right caudate nucleus**	**74**	**3.95**	**2.00**	**0.26**	**0.0261**	2.31 ± 0.76	0.0382 ± 0.0168		3.8	74	8.34	1.22	0.19	0.1119			
**Left caudate nucleus**	**76**	**4.51**	**2.19**	**0.25**	**0.0283**	2.80 ± 0.80	0.0410 ± 0.0184		4.1	76	8.72	1.05	0.17	0.1324			
**Right putamen**	78	3.24	1.15	0.14	0.2199					80	7.94	0.93	0.17	0.1287			
**Left putamen**	75	3.36	1.16	0.11	0.3382					**80**	**7.99**	**0.84**	**0.27**	**0.0142**	7.28 ± 0.30	0.0166 ± 0.0066	1.7
**Right pallidum**	74	3.39	1.56	0.00	0.9889					75	8.74	1.04	0.13	0.2482			
**Left pallidum**	68	3.53	1.43	−0.19	0.1160					77	9.13	1.08	0.02	0.8503			
**Right thalamus**	**77**	**3.60**	**1.02**	**0.25**	**0.0286**	2.82 ± 0.37	0.0183 ± 0.0082		1.8	**80**	**7.74**	**0.83**	**0.31**	**0.0054**	6.96 ± 0.29	0.0185 ± 0.0065	1.8
**Left thalamus**	77	3.53	1.25	0.12	0.3082					80	7.89	1.02	0.15	0.1920			

^a^*n* = number of sampled subjects. Values of several ROIs were not sampled for all subjects according to quality criteria; ^b^ in ratio to internal water and in institutional units for metabolites, intercept and slope are given as mean ± SE; ^c^ calculated in ratio to the values at age of 20 years and given in percentage per decade.

**Table 2:** Mean absolute Cramér–Rao lower bounds of mI, Gln, and Glu and associated standard deviations (SD).

Brain Region	mI		Gln		Glu	
	Mean	SD	Mean	SD	Mean	SD
Right caudate nucleus	6.07	2.02	13.23	7.21	13.69	5.40
Left caudate nucleus	6.27	2.55	10.23	8.06	13.07	5.35
Right putamen	5.33	1.05	9.46	6.04	10.71	3.02
Left putamen	4.86	0.97	10.57	6.30	11.07	4.22
Right pallidum	7.38	2.49	16.89	10.96	17.97	8.14
Left pallidum	7.63	1.96	14.29	10.94	18.09	7.30
Right thalamus	5.37	1.21	10.72	6.28	11.65	4.45
Left thalamus	5.02	1.17	11.01	6.81	11.15	4.48

**Table 3:** Correlations between measured Glu and Gln concentration at each brain region estimated with Pearson’s correlation test.

Brain Region	R	*p*	*n*
Right caudate nucleus	−0.51	<0.001	70
Left caudate nucleus	−0.57	<0.001	68
Right putamen	−0.59	<0.001	73
Left putamen	−0.53	<0.001	75
Right pallidum	−0.71	<0.001	71
Left pallidum	−0.54	<0.001	72
Right thalamus	−0.59	<0.001	73
Left thalamus	−0.40	<0.001	74

## Data Availability

The full results presented in this study are available on request from the corresponding author. The data are not publicly available due to local data protection policies of the University hospital.
